# Physical Properties and Biodegradability Evaluation of Vulcanized Epoxidized Natural Rubber/Thermoplastic Potato Starch Blends

**DOI:** 10.3390/ma15217478

**Published:** 2022-10-25

**Authors:** Zhejing Cai, Drahomír Čadek, Michaela Jindrová, Alena Kadeřábková, Antonín Kuta

**Affiliations:** Department of Polymers, University of Chemistry and Technology, Prague, Technická 5, 166 28 Praha, Czech Republic

**Keywords:** sustainable, thermoplastic starch, epoxidized natural rubber, accelerator, biodegradable

## Abstract

The sustainable material—thermoplastic potato starch (TPS)—was blended with modified natural rubber–epoxidized natural rubber (ENR). The poor mechanical properties of the ENR/TPS blends limited the application. Sulfur vulcanization is a common and economical method to improve the mechanical properties in the rubber industry. To fully understand the relationship between vulcanization systems and ENR/TPS blends and the sustainability of the developed material, the effects of a vulcanization accelerator (N-cyclohexylbenzothiazole-2-sulphenamide (CBS), 2-mercaptobenzothiazole (MBT), N-tert-butylbenzothiazole-2-sulphenamide (TBBS)) and a system type (conventional vulcanization (CV), semi-efficient vulcanization (SEV) and efficient vulcanization (EV)) on curing characteristics, mechanical and thermal properties, water absorption and biodegradability were systematically evaluated. The results indicate that vulcanization significantly improves the mechanical properties of ENR/TPS blends. The performance optimization of the CBS-CV vulcanization system is the best for improving the mechanical properties and reducing the water absorption. The CBS-CV curing system makes ENR/TPS less biodegradable (12–56% of mass loss) than other accelerators and systems. TBBS-CV makes the material more biodegradable (18–66% of mass loss). The low rubber content enables the rapid biodegradation of the vulcanized blend. This has implications for research on sustainable materials. The material can be applied for eco-friendly packaging and agricultural films, etc. The investigation on performance by using common accelerators and systems provides ideas for industries and research.

## 1. Introduction

The development of sustainable materials has always been the focus of attention of society and researchers. Thermoplastic starch (TPS) is one of the most attractive eco-friendly materials for short-life products. It is a material obtained by processing the starch of a granular structure with thermal and mechanical force in the presence of plasticizers [1]. In addition to biodegradability, TPS has other beneficial properties. It is a renewable and flexible material that can be easily used in different processes (e.g., injection molding, blow molding, compression molding or extrusion) with standard equipment used to manufacture synthetic polymers. The main shortcomings of TPS include its retrogradation and unsatisfactory mechanical properties, as well as high brittleness [2,3].

Epoxidized natural rubber (ENR), known as elastomer, is a form of modified natural rubber with better functional properties and a higher polarity for better miscibility with polar materials [4]. It has properties such as oil resistance, air impermeability and damping. Owing to the semi-polarity (i.e., ENR-50), its compatibility with TPS (polar) is higher than that of natural rubber (non-polar) and its mechanical properties are better than NR/TPS blends as well. However, the unvulcanized ENR/TPS blends still exhibit limited mechanical properties and a low rate of biodegradability [3].

Vulcanization (curing) transforms a thermoplastic rubber compound into a highly elastic product through physical and chemical reactions. The principle of vulcanization is the formation of chemical cross-links between the rubber chains, thus forming a 3D network [5]. Vulcanization can generally be classified according to curing agent into sulfur, peroxide, metal oxide and special systems [6,7,8,9]. Sulfur vulcanization is the most common method in the industry. Sulfur vulcanizates tend to exhibit greater tensile strength than peroxide vulcanizates, as well as better abrasion resistance and tear strength [10]. The main polymers vulcanized by sulfur are natural rubber, styrene–butadiene rubber, polybutadiene rubber or ethylene–propylene–diene rubber [11].

Some studies show the vulcanization behaviors and other physical properties of ENR-related or TPS-related and other polymers from different perspectives (Table 1). However, it is hard to find published studies which have systematically addressed the effects of different vulcanization accelerators and systems on the performance of ENR/TPS compounds.

In order to fully understand the relationship between vulcanization systems and ENR/TPS blends, in this study, we systematically evaluated the effect of vulcanization accelerators (i.e., N-cyclohexylbenzothiazole-2-sulphenamide (CBS), 2-mercaptobenzothiazole (MBT) and N-tert-butylbenzothiazole-2-sulphenamide (TBBS)) and accelerator/sulfur ratios (i.e., conventional vulcanization (CV), semi-efficient vulcanization (SEV) and efficient vulcanization (EV) systems) on the curing characteristics, mechanical and thermal properties and water absorption. In order to confirm that the properties of the material were improved by vulcanization, the unvulcanized samples were also measured as a control. These accelerators and systems are commonly applied in the industry. In order to further understand the sustainability of the developed material, the biodegradability was also studied.

## 2. Materials and Methods

### 2.1. Materials

Potato starch (PRIMA A, dry content 80.6%, 80% amylopectin) was purchased from Škrobárny Pelhřimov Ltd. (Pelhřimov, Czech Republic); epoxidized natural rubber (50% epoxidation) was obtained from Muang Mai Guthrie Public Co., Ltd. (Phuket, Thailand); glycerol and toluene, ZnSO_4_·7H_2_O were purchased from PENTA s.r.o (Prague, Czech Republic); NaNO_2_ was purchased from Lach-Ner s.r.o (Neratovice, Czech Republic); sulfur was supplied by Zolfindustria s.r.l. (Trecate, Italy); stearic acid was provided by Setuza a.s. (Ústí nad Labem, Czech Republic); ZnO was obtained from Brüggemann GmbH (Lemgo, Germany); N-cyclohexylbenzothiazole-2-sulphenamide (CBS) and 2-mercaptobenzothiazole (MBT) were purchased from Dalian Richon Chem Co., Ltd. (Dalian, China); N-tert-butylbenzothiazole-2-sulphenamide (TBBS) was purchased from Istrochem a.s. (Bratislava, Slovakia).

The preparation of ENR/TPS blends was performed according to the previous work [1]. The ENR/TPS compounds were prepared in an internal mixer (Brabender Plasti-Corder Lab-Station, Duisburg, Germany) at 90 °C and at a rotor speed at 100 rpm for 15 min. The formulations are described in Table 2.

**Figure 1:**
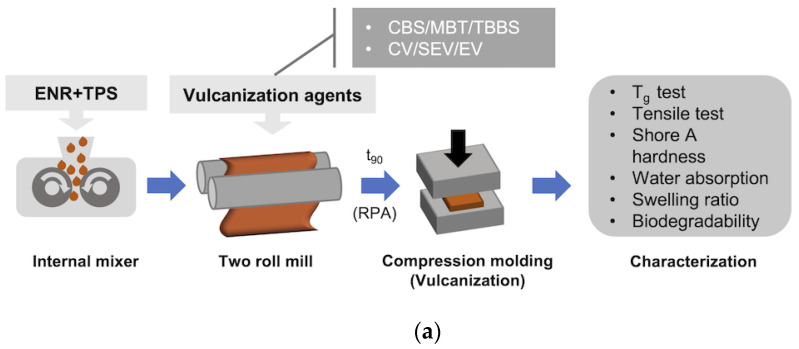

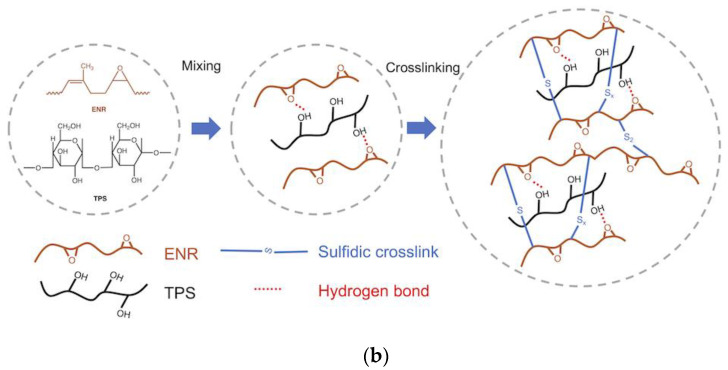
Schematic illustration of (**a**) the ENR/TPS blends’ preparation and vulcanization workflow; (**b**) the principle of ENR/TPS sulfidic crosslinking.

The prepared ENR/TPS mixtures were firstly masticated on a two-rolled mill at room temperature (BAŤA, Zlín, Czechoslovakia) and then the vulcanization agents were added and milled for 20 min. All milled mixtures were molded and vulcanized into a sheet (150 × 150 × 2 mm) by hydraulic press at 150 °C for optimal vulcanization time based on (t_90_) (Figure 1).

The vulcanization of the mixtures was divided into three groups by using 3 types of vulcanization accelerators (CBS, MBT and TBBS). The details of the accelerators are mentioned in Table 3. By comparing the tensile properties, the optimal accelerator will be used for the study of different vulcanization systems (CV, SEV and EV). The specific vulcanization formulations are shown in Table 4 and Table 5.

In the following text, the configuration “accelerator + ENR/TPS ratio + vulcanization system” is specified to indicate different samples. For example, “CBS/E30T70/CV” indicates that the ENR/TPS (30:70) blend was vulcanized with a CV curing system by using CBS as an accelerator. Uncured ENR/TPS blends were prepared with the same method (except vulcanization) for comparison.

### 2.2. Methods

#### 2.2.1. Vulcanization Characteristics

The curing characteristics of ENR containing different concentrations of TPS were determined (according to ASTM D 5289) [22] using an RPA 2000 (Alpha Technologies, Wilmington, NC, USA) rheometer with an oscillating rotor at 150 °C for 30 min. The optimal vulcanization time (t_90_) and scorch time (t_0.2_) were measured.

#### 2.2.2. Glass Transition Temperature

The glass transition temperature (T_g_) of vulcanized ENR/TPS blends and tan δ were performed by DMA (DX04 T, R.M. Electronic Measuring Instruments, Lázně Bohdaneč, Czech Republic). The measurement for the un-vulcanized samples was conducted for comparison. Samples cut into the dimensions of 40 × 8 × 2 mm were loaded with periodic bending at a constant amplitude of 500 mN, in a sinusoidal motion and the frequency of 1 Hz. The temperature mode of the measurement was from −93 to 100 °C and the heating rate was 2 °C/min.

#### 2.2.3. Tensile Properties

Tensile properties were measured according to ISO 37 standard using dumbbell-shaped specimens [23]. The test was carried out on an Instron Universal Testing Machine 3365 (Instron, Norwood, MA, USA) at a fixed crosshead speed of 500 mm/min at room temperature. The reported values were the average of 6 replicates for the property test of each material.

#### 2.2.4. Hardness

The Shore A hardness of the samples was measured with a hardness tester (HPE III Bareiss, Oberdischingen, Germany) according to the ISO 48-4 test method [24]. The thickness of each specimen was 6 mm.

#### 2.2.5. Water Absorption

The water absorption was characterized by placing the specimens (5 × 5 × 5 mm) in two different environments with relative air humidity based on the ASTM E 104 standard [25]. The environment of high relative air humidity (90%) and low relative air humidity (66%) was constructed by, respectively, placing a saturated ZnSO_4_·7H_2_O solution and a saturated NaNO_2_ solution in two independent airtight desiccators at a constant room temperature (app. 23 °C). After one week, the samples were weighed and then dried in a desiccator with silica gel to a constant weight (app. 2 weeks). The water absorption was calculated by the following equation:

(1) $$Water\;absorption\;\left(\%\right)=\frac{\left(w_{2}-w_{1}\right)}{w_{1}}\times 100$$

where *w*_2_ is the mass [g] of the test specimen in the humidified condition; *w*_1_ is the mass [g] of the test specimen in the dried state.

#### 2.2.6. Swelling Ratio

The swelling ratio of the samples was determined by the equilibrium swelling method (ISO 1817) [26]. A sample weighing about 0.4–0.5 g (30 × 5 × 2 mm) was cut from the molded rubber sample. The samples were immersed in pure toluene at room temperature for 1 week [27]. After that, the used toluene solvent was replaced by fresh toluene solvent for 1 more week. The samples were taken out, their surface was wiped off and the samples’ weight was determined. This was followed by drying the samples to a constant weight for 2 weeks at room temperature. The swelling ratio *SR* of the sample was calculated from the following equation:

(2) $$SR\;\left(\%\right)=\frac{m_{s}-m_{d}}{m_{d}}\times 100$$

*m_d_* is the weight of the sample before swelling, and *m_s_* is the weight of the swollen sample. The swelling ratio is an indicator of the degree of crosslink: the smaller the ratio, the higher the degree of crosslinking. A swelling test is performed to observe the filler–rubber matrix interaction. The swelling ratio is the quantity of solvent uptake per weight of rubber [28].

#### 2.2.7. Biodegradability by Composting Test

**Figure 2:**
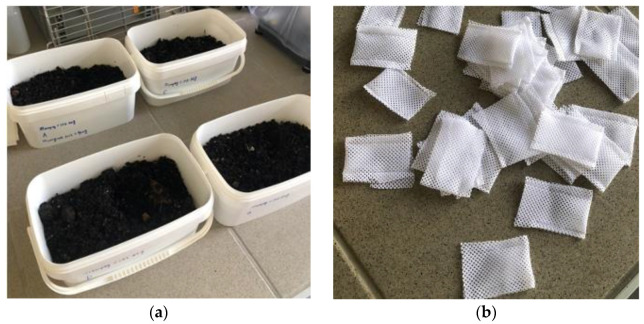
(**a**) Compost soil. (**b**) Polyester fabric bags.

The biodegradation of the compostable materials was tested in a controlled experimental environment. The experimental setup for the laboratory experiment is based upon procedures outlined in ASTM D5338 [29]. Each of the compostable materials was added to compost soil in a 2.5 L cuboid plastic container and placed in an oven maintained at 58 °C. The containers have a plastic seal on the top. Aeration holes were drilled at the top of the vessel. The samples were buried under 900 g of mature soil compost in the vessel. The mature compost, when 2–3 months old, had a pH of 8.7, an ash content of 35% and a carbon/nitrogen (C/N) ratio of 10. The samples were cut into 1 × 1 cm sheet specimens, dried in a desiccator in a silica gel environment and weighed. Four specimens were prepared for each sample and marked as A, B, C, D. Different marks represent the difference in time to take out the sample. They were then sealed into polyester fabric bags with holes, (Figure 2b) and placed into the compost soil (Figure 2a). The compost was moistened regularly to maintain the moisture around 50%. After one week, the compost samples of the first group (A) were removed, dried in a desiccator and weighed. The biodegradability of the material was determined by composting according to the equation:

(3) $$BIC=\frac{m_{0}-m_{1}}{m_{0}}\cdot 100\%$$

where *BIC*—biodegradability in compost, *m*_0_—mass of the test specimen before decomposition (g), *m*_1_—mass of the test specimen after decomposition in compost and drying (g).

The samples of set B were removed after 2 weeks, the samples of set C were taken out after 4 weeks and the samples of set D were removed after 5 weeks from the compost.

## 3. Results and Discussion

### 3.1. Curing Behavior

**Figure 3:**
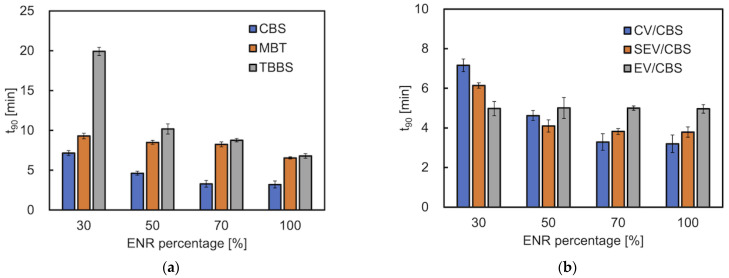

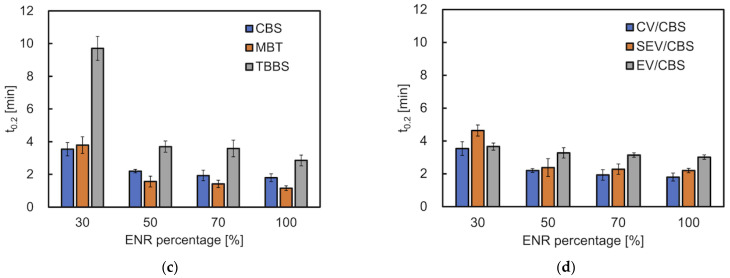
The optimal vulcanization time t_90_ and scorch time t_0_._2_ of ENR/TPS in different ratios: (**a**,**c**) different cure accelerators (CBS, MBT, TBBS); (**b**,**d**) different cure systems with CBS (CV/CBS, SEV/CBS, EV/CBS).

**Figure 4:**
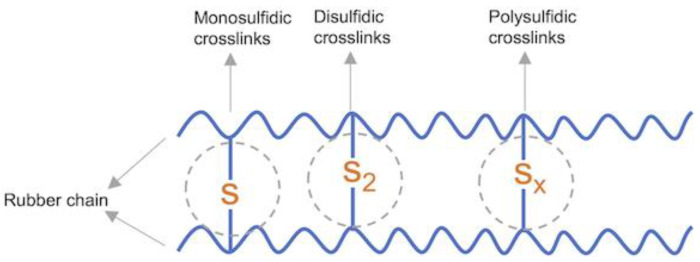
Typical chemical bonds (monosulfidic, disulficdic and polysulfidic crosslinks) present in the sulfur vulcanized ENR.

The optimal vulcanization time (t_90_) of the investigated materials significantly depends on the type of accelerator and the ratio of accelerator to sulfur (Figure 3a,b). It is the time it takes for the material to torque to 90% of its maximum value. It is related to the time it takes for the vulcanized rubber to reach its optimum properties [30]. In general, with ENR content increased, the t_90_ decreased. This showed that the high content of TPS hinders or slows down the vulcanization crosslinking of ENR. However, the ENR content in the blends using the MBT-CV curing system had little effect on t_90_ (8.3–9.3 min). This mainly depends on the nature of the vulcanization accelerator. Regarding the types of accelerators (Figure 3a), CBS exhibits the shortest t_90_ (3.3–7.2 min), then followed by MBT. Blends with TBBS exhibit the longest t_90_ (8.8–19.9 min). This indicates that the crosslinking of the mixtures with systems containing CBS strongly promotes the formation of sulfidic crosslinks (Figure 4). Comparing different vulcanization systems (Figure 3b), the t_90_ of CBS/E70T30/CV has been the lowest (value 3.3 ± 0.4 min).

Scorch time (t_0.2_) is the maximum length of time for which a rubber compound can be worked at a given temperature before curing begins [19] (Figure 3c,d). The t_0.2_ of mixtures containing TBBS was the longest (values 3.6–9.7 min) compared with the other two accelerators. The ENR content has little effect on the t_0_._2_ of the EV curing system (values 3.1–3.7 min).

### 3.2. Glass Transition Temperature (T_g_)

**Figure 5:**
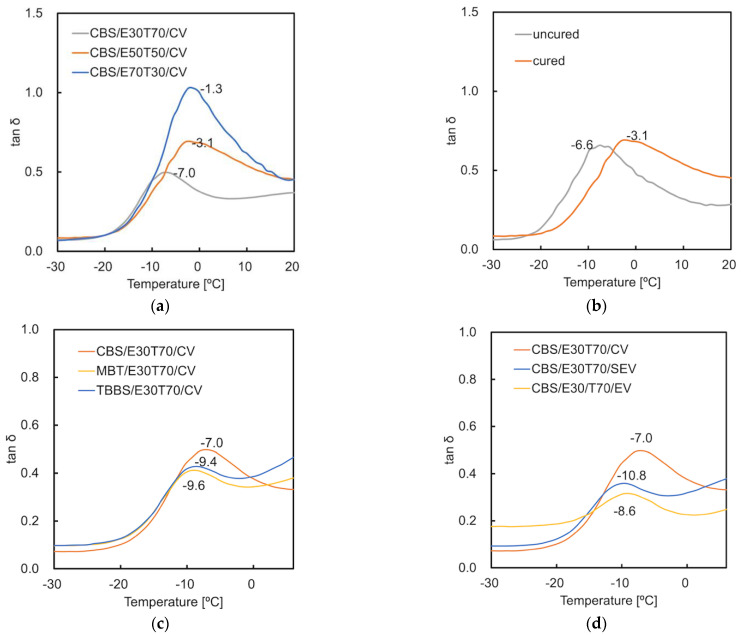
Effect of (**a**) ENR content (30 wt.%, 50 wt.%, 70 wt.%); (**b**) curing (CBS/E50T50/CV); (**c**) vulcanization accelerator types (CBS, MBT, TBBS); (**d**) sulfur/accelerator ratio (CV/CBS, SEV/CBS, EV/CBS) on the loss tangents as function of temperature.

Further research on glass transition temperature (T_g_) was performed on the samples which showed better mechanical properties (Figure 5). It is an important parameter for identifying polymer materials. The T_g_ value is the temperature at which an amorphous polymer changes from hard to soft [31]. For every 20 wt.% increase in ENR content, the T_g_ of the studied material increases by around 4 °C (−7.0, −3.1, −1.3 °C) (Figure 5a). A similar trend was confirmed by V. S. Mathew et al. [32]. The vulcanization shifted the T_g_ of CBS/E50T50/CV from −6.6 to −3.1 °C. This can be explained by the reduced molecular chain mobility due to the crosslink created by vulcanization. A similar phenomenon was mentioned by Mansilla, M. A. et al. [33]. CBS in CBS/E30T70/CV shows the highest T_g_ (−7.0 °C) compared with the other two accelerators. The CV curing system leads to the material with the highest T_g_ compared with CBS/E30T70/EV (−8.6 °C) and CBS/E30T70/SEV (−10.8 °C) (Figure 5c,d). This phenomenon further proved the higher crosslinking level in the CBS-CV curing system.

### 3.3. Tensile Properties

**Figure 6:**
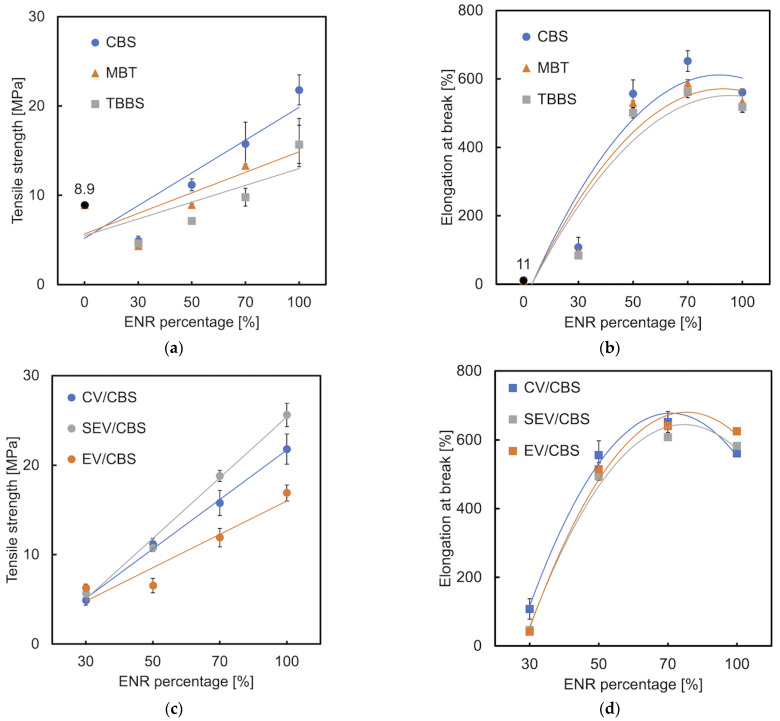

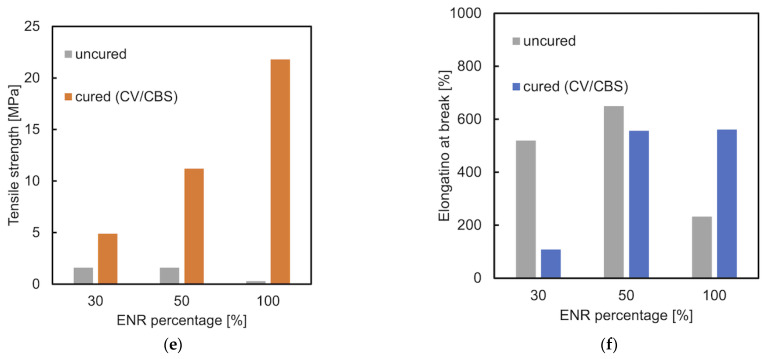
Tensile strength and elongation at break of ENR/TPS in different ratios and (**a**,**b**) different accelerator types; (**c**,**d**) different cure systems; (**e**,**f**) cured and uncured samples.

The influence of the cure-type accelerators and vulcanization systems on the tensile properties of the developed materials is shown in Figure 6. Usually, tensile strength increases with crosslink density and elongation at break is inversely proportional to it. This was confirmed by L. González et al. [34]. Comparing the tensile strength of the investigated blends with different curing accelerators (CV system), materials with CBS proved to have the highest value (Figure 6a). This indicates the highest crosslinking of these samples which was confirmed by the swelling ratio in Section 3.6. Unexpectedly, an increasing trend is shown in the elongation at break (Figure 6b). This can be explained by the binding of ENR to TPS which breaks the hydrogen bonds in the starch structure. The higher the ENR content in the mixture, the more hydrogen bonds are broken, which results in the increases in elongation at break. This also explains our previous research [3]. The materials (CBS-CV) which contain 50 wt.% ENR and higher, show a significantly higher tensile strength than TPS alone. All the investigated ENR/TPS blends show much higher values of elongation at break than those of TPS. Regarding the influence of different sulfur/accelerator ratios on the tensile strength, the samples cured by the CBS-SEV systems have the highest values, followed by the CV system. This is because due to the higher concentration of accelerator in the SEV system, a reaction such as either desulphurisation or decomposition may occur. Desulphurisation results in mono and disulfide linkages, whereas decomposition leads to cyclic sulfides and dienes. In accordance with [35], the higher the concentration of mono and disulfide crosslinks, the higher the tensile strength of the SEV compared to that of the CV system containing the higher concentration of polysulfide crosslinks. However, based on our previous experimental results, vulcanizates with polysulfidic crosslinks have higher tensile strength and elongation at break than those with monosulfidic ones. The noticeable improvement of tensile strength can be observed in the comparison of all tested cured (CBS-CV) and uncured samples (Figure 6e). The elongation at break of the cured ENR/TPS (30/70) shows a significantly lower value (110%) than the uncured one (520%). This may be explained by the stronger influence of decrease in crosslink density (Figure 6f). The values of cured (560%) and uncured (650%) samples of E50T50 are very close.

### 3.4. Shore A Hardness

**Figure 7:**
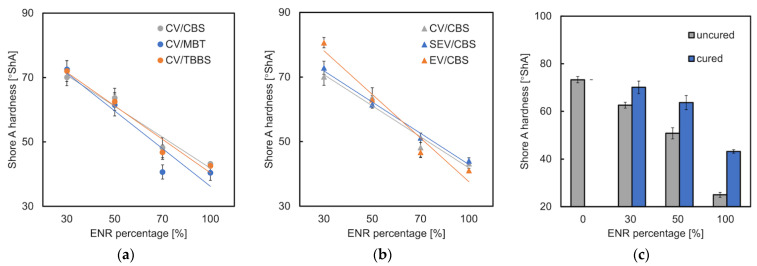
Shore A hardness of ENR/TPS in different ratios and (**a**) different accelerator types; (**b**) different cure systems; (**c**) cured (CBS/CV) and uncured samples.

The shore A hardness of the samples was presented in Figure 7. Comparing the hardness of the samples containing different accelerator types (CV system), there is not significant difference for the cured E30T70 samples (70–73 °ShA) (Figure 7a). The softest sample is MBT/E70T30/CV (41 °ShA). CBS leads to a higher hardness value in “high ENR content” (ENR% ≥ 50%) samples than in the samples containing MBT and TBBS. This can be explained by more vulcanization and higher amount of crosslinks. Regarding various curing systems (Figure 7b), the hardest (81 °ShA) and softest (47 °ShA) samples are both prepared by EV vulcanization. The CV system makes the cured E30T70 samples softer than the two other cure systems (EV, SEV) due to a higher amount of polysulfidic crosslinks, which was mentioned in the tensile Section 3.3. The hardness comparison of the cured and uncured samples is shown in Figure 7c. The hardness of the cured (CBS-CV) samples is significantly higher than that of the uncured samples. This further demonstrates that crosslinking occurs efficiently.

### 3.5. Water Absorption

**Figure 8:**
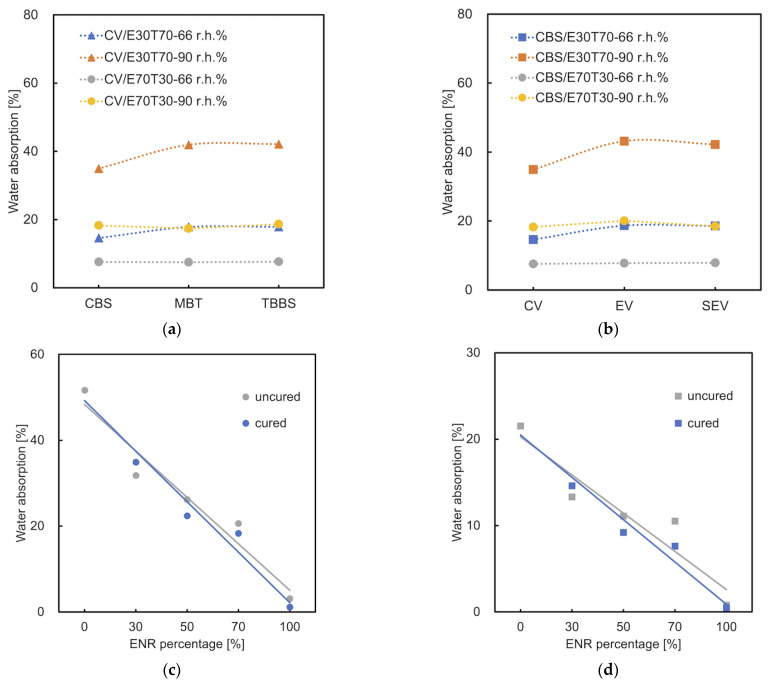
Water absorption of ENR/TPS in different ratios and (**a**) different accelerator types (CV system); (**b**) different cure systems (CBS); (**c**) cured (CBS/CV) and uncured samples in 90 r.h.%; (**d**) cured (CBS/CV) and uncured samples in 66 r.h.%.

Water absorption was measured for the samples which showed better mechanical properties. It is reasonable that higher levels of ENR% result in a lower water uptake of the mixture, regardless of environmental humidity. Comparing different accelerator types (CV system), the CBS samples show the lowest water absorption (Figure 8a). This proves that the most efficient crosslinking is achieved by using CBS. As the crosslinking density is increased, the water diffusion decreases and the slowdown in diffusion is more severe at the polymer−water interface. The water diffusion at various crosslinking densities correlates with the water–hydrogen bonding dynamics [36]. However, the number of hydrogen bonds/hydrophilic groups open to interaction with water affects this hydrophilicity. The samples vulcanized by the CV system (CBS accelerator) have a lower water absorption than those of the SEV and EV systems (Figure 8b). This is because the CV system contains the highest amount of sulfur, which was pointed out by Ismail, H., Salmah and M. Nasir [37]. Furthermore, a higher concentration of crosslinks in the CV system, which was proved by means of the swelling ratio, as seen in Section 3.6, confirms the low water absorption. It can be observed that the cured sample CBS/E30T70/CV shows higher water absorption than the uncured blend (Figure 8c,d). When ENR% ≥ 50%, the water absorption of cured materials is lower than the uncured samples. This is consistent with the results of the tensile test and can be explained as there are more hydrogen bonds broken than effective crosslinks created in the low ENR% mixture. The breaking of hydrogen bonds results in higher hydrophilicity [38,39].

### 3.6. Swelling Ratio

**Figure 9:**
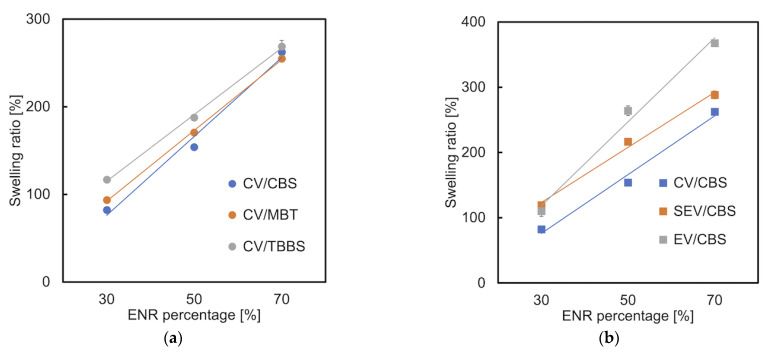
Swelling ratio of ENR/TPS in different amount ratios and (**a**) different accelerator types (CV system); (**b**) different cure systems (CBS).

The swelling ratio of a polymer is generally affected by its crosslink density. That is, the lower the crosslink density, the higher their ability to accommodate more solvent molecules [40,41,42]. The increase in swelling ratio corresponded with the increase in ENR loading in toluene. The swelling ratio of pure TPS is −0.4% ± 0.05, which indicates an un-swollen state. Generally, the ENR/TPS blends cured by using a CBS accelerator (Figure 9a) and the CV curing system (Figure 9b) exhibit the lowest swelling ratio. The lower value indicates the higher degree of crosslinking [28]. The tensile strength results in Section 3.3 show that the samples vulcanized with the CBS accelerator showed the highest tensile strength and elongation at break, also demonstrating the higher degree of crosslinking. It was also fascinating to see a similar trend in the DMA results because the “CBS samples” had a higher tan δ peak than “MBT and TBBS samples”. This indicates that CBS makes the blends have a narrower transition from a glassy state to a rubbery state. The higher crosslinking degree in the CV system’s blends indicates a higher crosslinking efficiency than the two other systems.

### 3.7. Composting Test

**Figure 10:**
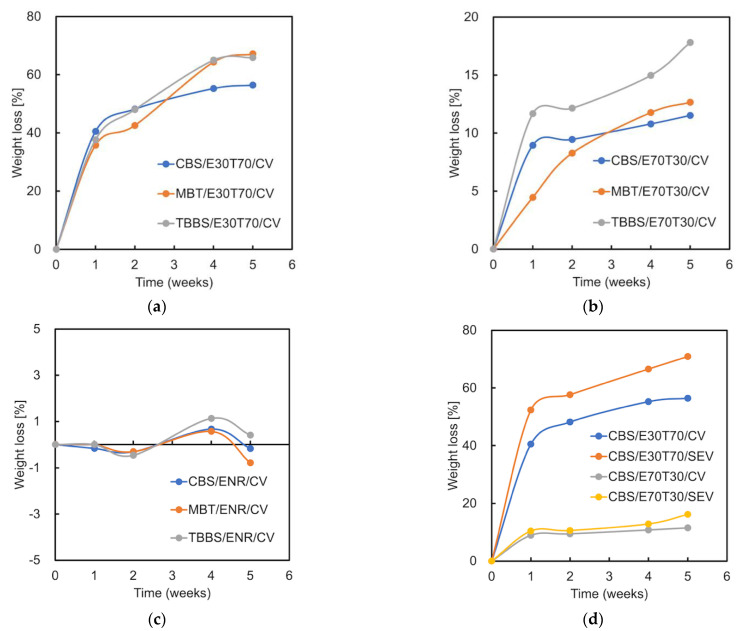
Mass loss during composting degradation test of (**a**) vulcanized ENR/TPS blends with 30% ENR; (**b**) vulcanized ENR/TPS blends with 70% ENR; (**c**) vulcanized pure ENR with different accelerators; and (**d**) vulcanized ENR/TPS blends prepared by various curing systems.

The weight loss observed among the vulcanized ENR/TPS blends during the degradation process is depicted in Figure 10. The weight loss that had been recorded ascertains that the degradation process did take place in the samples due to the presence of moisture and microorganisms in the soil. The weight loss further caused some changes to the surface morphology of the samples. In Figure 10d, the mass loss percentage of the cured E30T70 samples is significantly higher than for the cured E70T30 samples, which confirmed that the main biodegraded part is TPS. The samples cured by the SEV system show a higher mass loss percentage than those of the CV system, which indicates better biodegradability. This proves that better biodegradability also corresponds to lower crosslinking efficiency in the SEV system than in the CV system, which was described in the swelling ratio section. During the first two weeks, the MBT samples exhibited a lower weight loss (degradation rate) compared to other two. This is due to the higher ENR swelling in the soil moisture (Figure 10c). The swelling behavior can be found in the previous research as well [3]. Furthermore, the water absorption of the MBT-CV samples shows a higher value than that of the CBS-CV samples (Figure 8a). The significant weight loss of pure cured ENR after 4 weeks (Figure 10c) can be explained by the loss of fragments and small particles from the surface. The TBBS/E70T30/CV shows the highest mass loss (biodegradation rate) due to the poor crosslinking efficiency (Figure 10b). Low-chain network density accelerates the decomposition. After 4 weeks, the samples showed a lower biodegradation rate, except for TBBS/E70T30/CV. At the 5th week, the mass loss of CBS/E30T70/CV was 56.4%, which is less than that of the SEV system sample (70.8%). The same trend for the CBS/E70T30 is observed: 11.5% for the CV sample and 16.2% for the SEV sample. This further indicates that the CBS samples exhibit the lowest mass loss compared to the other two due to the higher crosslinking degree.

## 4. Conclusions

This study is focused on comparing the effects of different vulcanization accelerator types and systems on the mechanical properties, thermal properties, water absorption and biodegradability of hybrid blends of epoxidized natural rubber and thermoplastic potato starch. It is clearly noted that vulcanization significantly improves the mechanical properties of ENR/TPS blends. The performance optimization of the CBS accelerator is better than that of MBT and TBBS. CV vulcanization systems are better than SEV and EV for improving mechanical properties. When vulcanization is applied to blends with an ENR content of 50% or higher, water absorption is effectively reduced (compared to unvulcanized blends). The CBS-CV system is the most effective for water absorption reduction. Vulcanization increases the hardness of the material, with CBS-CV increasing it the most. The CBS-CV curing system makes ENR/TPS less biodegradable than other accelerators and systems. TBBS-CV makes the material highly biodegradable. The low ENR% content enables the rapid biodegradation of the vulcanized blend. This has implications for research on sustainable materials. The material can be used for environmentally friendly packaging or agricultural films, etc. Further research on different preparation methods to reduce energy consumption will be carried out in the future.

## Figures and Tables

**Table 1:** The summary of the literature search related to vulcanized rubber compounds of ENR and TPS.

Polymers	Purposes	Results	Reference
ENR/PP ^a^ blends	Effect of vulcanization system (sulfur, peroxide and a mixture of sulfur and peroxide) on properties.	The mechanical properties have been improved by the mixture of sulfur and peroxide cure system.	[12]
ENR	(1) The effect of accelerator/sulfur ratio on the scorch time; (2) Cure properties of unaccelerated sulfur vulcanization; (3) The effect of cure systems on cure properties, physical properties and thermal aging.	(1) t_0_._2_ ^c^: CV > SEV > EV; (2) t_0_._2_: ENR-25 > ENR-50; (3) t_90_ ^d^: EV > CV; crosslink density: CV > EV; SEV shows the highest thermal stability.	[13,14,15]
ENR/NR	Tensile properties.	A maximum tensile strength and elongation at break at 50% ENR for both ENR-25 /NR and ENR-50/NR blends.	[16]
ENR-30/XSBR ^b^ blends	Preparation and characterization.	t_0_._2_ and t_90_: blends > ENR-30 300% modulus increased, tensile strength and elongation at break decreased.	[17]
HDPE ^e^/NR/TPS blends	Effects of dynamic curing on the physical, mechanical and morphological properties.	Good dispersity, improved tensile strength.	[18]
Reclaimed ground tire rubber	Curing, mechanical and thermal properties.	TMTD sample—highest crosslink density.	[19]
ENR/PAA ^f^	Physical properties.	The moisture content and absorption were increased, the elongation at break and toluene resistance were improved.	[20]
TPS/Polylactic acid/NR	The effects of dynamic vulcanization on the vulcanization system.	The addition of HVA-2 ^g^ and DCP ^h^ curatives showed an increase in crosslinking density and better tensile properties. Both of the vulcanization systems delayed biodegradability.	[21]

^a^—polypropylene; ^b^—carboxylated styrene butadiene rubber; ^c^—scorch time; ^d^—optimal vulcanization time; ^e^—high-density polyethylene; ^f^—plant amino acid; ^g^—N, N′-m-phenylene bismaleimide; ^h^—dicumyl peroxide.

**Table 2:** Formulations used for preparation of ENR/TPS blends.

Sample	ENR [wt. %]	TPS [wt. %]
1	0	100
2	30	70
3	50	50
4	70	30
5	100	0

**Table 3:** The accelerators used in this work.

Accelerator	Chemical Name	Chemical Structure
CBS	N-cyclohexylbenzothiazole-2-sulphenamide	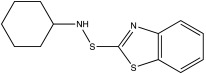
MBT	2-mercaptobenzothiazole	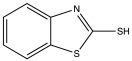
TBBS	N-tert-butylbenzothiazole-2-sulphenamide	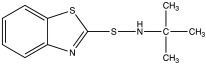

**Table 4:** Vulcanization formulas of ENR/TPS blends with 3 types of accelerators.

	I [phr]	II [phr]	III [phr]
ENR/TPS	100	100	100
ZnO	5	5	5
Stearic acid	2	2	2
S	2.5	2.5	2.5
CBS	1	0	0
MBT	0	1	0
TBBS	0	0	1

**Table 5:** Vulcanization formulas of ENR/TPS blends with 3 cure systems.

	CV [phr]	SEV [phr]	EV [phr]
ENR/TPS	100	100	100
ZnO	5	5	5
Stearic acid	2	2	2
S	2.5	1.7	0.8
CBS	1	2	3.5

## Data Availability

This will be made available upon request through the corresponding author.
